# Suppressing the Initial Growth of Sidewall GaN by Modifying Micron-Sized Patterned Sapphire Substrate with H_3_PO_4_-Based Etchant

**DOI:** 10.3390/mi9120622

**Published:** 2018-11-26

**Authors:** Wen-Yang Hsu, Yuan-Chi Lian, Pei-Yu Wu, Wei-Min Yong, Jinn-Kong Sheu, Kun-Lin Lin, YewChung Sermon Wu

**Affiliations:** 1 Department of Materials Science and Engineering, National Chaio Tung University, Hsinchu 300, Taiwan; j06900397@hotmail.com (W.-Y.H.); kiu741@yahoo.com.tw (Y.-C.L.); a3062123@hotmail.com (P.-Y.W.); cowbell506@yahoo.com.tw (W.-M.Y.); 2 Department of Photonics, National Cheng Kung University, Tainan City 701, Taiwan; jksheu@mail.ncku.edu.tw; 3 National Nano Device Laboratories, Hsinchu300, Taiwan; kllin@narlabs.org.tw

**Keywords:** micron-sized patterned sapphire substrate, growth of GaN, sidewall GaN

## Abstract

Micron-sized patterned sapphire substrates (PSS) are used to improve the performance of GaN-based light-emitting diodes (LEDs). However, the growth of GaN is initiated not only from the bottom c-plane but also from the sidewall of the micron-sized patterns. Therefore, the coalescence of these GaN crystals creates irregular voids. In this study, two kinds of nucleation layers (NL)—ex-situ AlN NL and in-situ GaN NL—were used, and the growth of sidewall GaN was successfully suppressed in both systems by modifying the micron-sized PSS surface.

## 1. Introduction

High-brightness GaN-based light-emitting diodes (LEDs) are used in a wide variety of applications [1,2]. However, a GaN epitaxial layer usually contains several defects due to the large lattice mismatch and the thermal expansion coefficient difference between GaN and sapphire.

An AlN (or GaN) nucleation layer (NL) is commonly introduced prior to growth of GaN epilayer to overcome this lattice mismatch problem [3,4]. Moreover, micron-sized patterned sapphire substrates (PSS) have been successfully used to reduce these defects and enhance the performance of LEDs [5,6,7,8,9,10,11,12].

When PSS are used, the growth of GaN is initiated not only from the bottom c-plane but also from the sidewall of the micron-sized patterns [13,14,15,16]. As the growth time increases, irregular voids are created during the coalescence of these GaN crystals [17].

A GaN NL is usually deposited by metal–organic chemical vapor deposition (MOCVD), and it is called in-situ GaN NL. An AlN NL can be deposited either by MOCVD or sputtered physical vapor deposition (PVD), and they are generally called in-situ AlN NL and ex-situ AlN NL, respectively. It has been found that ex-situ sputtered AlN NL has better GaN quality than in-situ GaN NL and in-situ AlN NL [15,18].

In this study, sulfuric–phosphoric acid was used to modify the micron-sized patterns in order to suppress the growth of sidewall GaN. The effect of this modification on the growth mechanism of GaN was also investigated.

## 2. Materials and Methods

**Figure 1:**
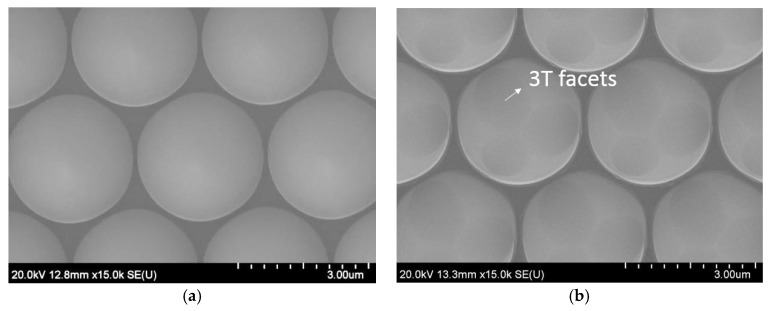
SEM images of (**a**) RPSS (regular patterned sapphire substrates) and (**b**) PSSE (RPSS etched in sulfuric–phosphoric acid).

In this study, commercial dry etching c-plane micron-sized PSS (2.8 μm width and 0.2 μm spacing) was modified. As shown in Figure 1, two kinds of PSS samples were used to investigate the effect of modification of micron-sized PSS patterns on the GaN growth mechanism: (1) RPSS (regular PSS without etching) and (2) PSSE (RPSS etched in sulfuric–phosphoric acid (ratio 3:1) at 270 °C for 30 s). As shown in Figure 1b, 3T {1$\bar{1}$05} facets were observed on the pattern of PSSE [19,20,21,22,23].

Two kinds of nucleation layers (NL) were used: (1) ex-situ AlN NL and (2) in-situ GaN NL. To fabricate ex-situ AlN NL, 40 nm AlN was deposited by RF-sputter system using Al target in N_2_ gas at 650 °C. As for the in-situ GaN NL, an in-situ 25-nm-thick low-temperature GaN layer was deposited at 550 °C by MOCVD.

As shown in Table 1, four kinds of micron-sized PSS samples were then used to investigate the effect of modification of PSS patterns on the GaN growth mechanism: (1) AlNR (RPSS with AlN NL); (2) AlNE (PSSE with AlN NL); (3) GaNR (RPSS with GaN NL); and (4) GaNE (PSSE with GaN NL).

To investigate the GaN epitaxial behavior, high-temperature undoped GaN (HTU-GaN) was grown by MOCVD at 1060 °C with chamber pressure of 200 torr (26,664 Pa) for 2 min.

## 3. Results

**Figure 2:**
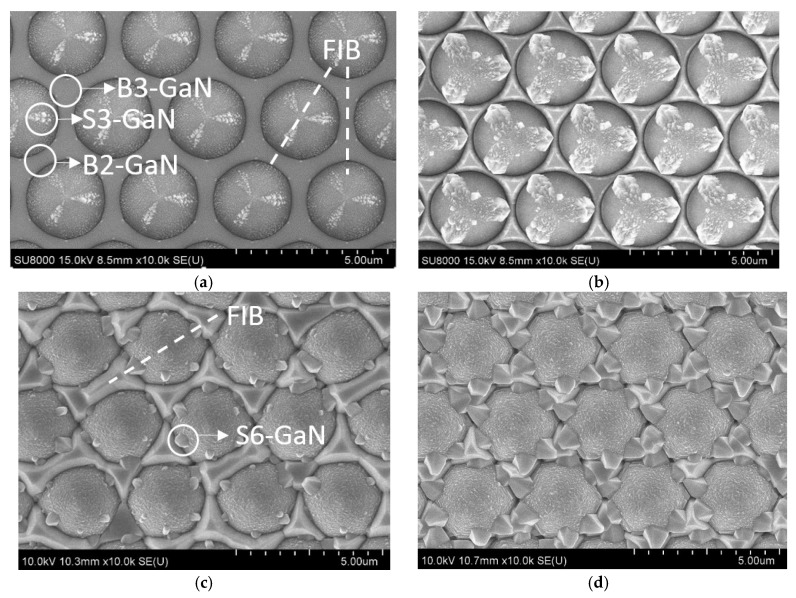
SEM images of GaN grown on (**a**) AlNR, (**b**) AlNE, (**c**) GaNR, and (**d**) GaNE.

**Figure 3:**
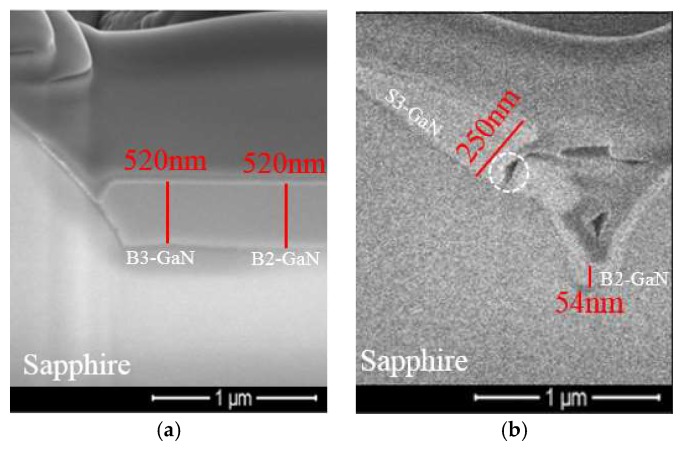

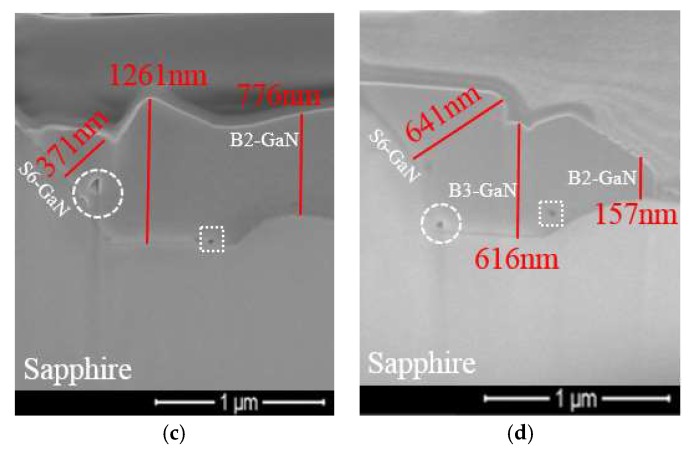
Cross-sectional SEM images from Figure 2. (**a**) AlNR, (**b**) AlNE, (**c**) GaNR, and (**d**) GaNE.

Figure 2 shows the surface morphologies of micron-sized PSS after GaN was grown. The morphologies of bottom GaN (B-GaN) and sidewall GaN (S-GaN) were different. There were two kinds of B-GaN: (1) B3-GaN (GaN grown among three micron-sized patterns) and (2) B2-GaN (GaN grown between two patterns). Two kinds of S-GaN were found: (1) S3-GaN (with AlN as NL; Figure 2a) and (2) S6-GaN (with GaN as NL; Figure 2c). To measure the thicknesses of B-GaN and S-GaN, cross-sectional SEM was carried out by focused ion beam (FIB) cutting along the dash lines as shown in Figure 2a,c. Some of the related images are shown in Figure 3, and the measured maximum thicknesses are summarized in Table 2.

## 4. Discussion

A simple treatment of the MOCVD thin-film growth kinetic involves mass transport and reaction [24,25]. It is reasonable to assume that the mass transport was the same for all the samples as GaN was grown in the same conditions.

In a reaction between A and B to give products C and D, the following applies according to the balance equation:

a A + b B → c C + d D(1)

The reaction is related to the reactant concentrations in the following way:

Rate = K [A]^x^[B]^y^ (2)

where K is the rate constant; the numbers x and y are partial orders of reaction.

In this case, there were four surface reaction constants: (1) sidewall with AlN (*K_SAlN_*), (2) bottom with AlN (*K_BAlN_*), (3) sidewall with GaN (*K_SGaN_*), and (4) bottom with GaN (*K_BGaN_*).

### 4.1. Ex-Situ AlN as NL

When ex-situ AlN NL was used, as shown in Figure 2a, two kinds of GaN were found on AlNR. B-GaN was initiated from the bottom c-plane as expected, while S-GaN (S3-GaN) was from sidewall surfaces, which has been reported earlier [14,15,26,27]. Both B-GaN and S3-GaN were Wurtzite structures. The orientation relationship between GaN (including B-GaN and S3-GaN) and sapphire was established as (0001)_GaN_ // (0001) sapphire and [1$\bar{1}$00]_GaN_ // [11$\bar{2}$0]_sapphire_.

As shown in Figure 3a, no void was found among GaN crystals as there was no coalescence yet between S-GaN and B-GaN.

Table 2 and Figure 3a show that the maximum thicknesses of B3-GaN (H_B3-GaN_) and B2-GaN (H_B2-GaN_) of AlNR were around 520 nm, which was much thicker than that of S3-GaN (H_S3-GaN_, 74 nm), indicating that *K_BAlN_* was much greater than *K_S3AlN_* [26]. 

However, with the modification of PSS patterns (AlNE), H_S3-GaN_ of AlNE did not diminish but increased. As shown in Figure 2b and Figure 3b, and Table 2, compared with AlNR, the H_S3-GaN_ of AlNE increased from 74 to 250 nm. At the same time, H_B3-GaN_ decreased from 520 to 136 nm, and H_B2-GaN_ decreased from 520 to 54 nm.

Moreover, as shown in Figure 3b, irregular voids (circled with dashed lines) were observed between S3-GaN and B2-GaN. These voids were created during the coalescence of GaN crystals [17].

This observation suggested that instead of reducing the reaction constant of S3-GaN (*K_S3AlN_*), modification of PSS patterns (AlNE) enhanced *K_SAlN_* and reduced *K_BAlN_*. As *K_BAlN_* should be a constant, we believe this *K_BAlN_* reduction should have been caused by the change in the area of the bottom c-plane.

**Figure 4:**
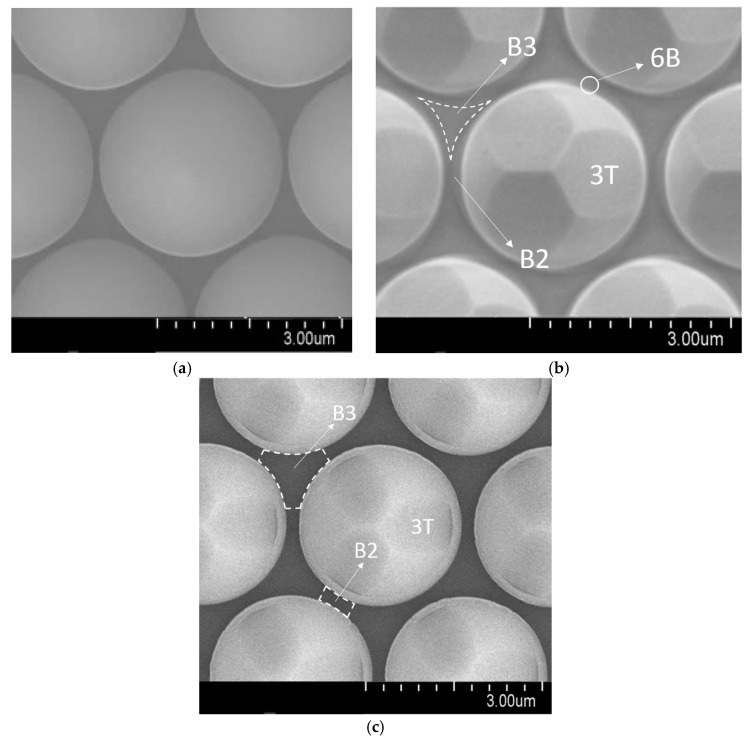
The high magnification SEM images of (**a**) RPSS, (**b**) PSSE, and (**c**) PSSO.

Figure 4 is the high magnifications of (a) RPSS and (b) PSSE. In addition to sidewall facets, an extra six 6B {3$\bar{4}$17} facets were found on the bottom of patterns of PSSE [28], as shown in Figure 4b. The appearance of 6B facets reduced the bottom c-plane fraction as determined by estimating the B3 (B2) vs. total area on the SEM images. 

Compared with RPSS, B3 fraction of PSSE reduced from 18% to 12%, while B2 fraction reduced from 3% to 0 %. This reduction of bottom c-plane made epitaxy of GaN film on PSSE very difficult [29]. Consequently, it appeared that the *K_BAlN_* of AlNE was much less than that of AlNR.

In addition, compared with AlNR, H_S3-GaN_ of AlNE increased from 74 to 250 nm, as shown in Figure 2b and Figure 3b and Table 2. This is because the consuming of the reactants in front of the bottom c-plane can affect reactant concentrations in front of the sidewall. As shown in Figure 4, the distance between the sidewall and the bottom c-plane was only around 1 µm. As the growth rate of AlNE was much smaller than that of AlNR, the formation of B-GaN of AlNE would consume only a small portion of the reactants. As a result, the reactant concentrations in front of the sidewall of AlNE were increased. Consequently, H_S3-GaN_ of AlNE was thicker than that of AlNR.

### 4.2. In-Situ GaN as NL

When in-situ GaN NL was used, as shown in Figure 2c, two kinds of GaN were found on AlNR: (1) B-GaN and (2) S6-GaN [13,14,15,16]. They were both Wurtzite structures, and the orientation relationship between GaN and sapphire was established as (0001)_GaN_ // (0001) sapphire and [1$\bar{1}$00]_GaN_ // [11$\bar{1}$0]_sapphire_.

Table 2 and Figure 3c show that H_B3-GaN_ and H_B2-GaN_ of GaNR were 1261 nm and 776 nm, respectively. Their thicknesses were much greater than H_S6-GaN_ (371 nm) [14,27]. In other words, *K_BGaN_* was much greater than *K_S6GaN_*.

We also found that modification of PSS patterns (PSSE) did not diminish the growth of sidewall GaN (S6-GaN). Compared with GaNR, H_S6-GaN_ of GaNE increased from 371 to 614 nm. At the same time, H_B3-GaN_ decreased from 1261 to 616 nm, and H_B2-GaN_ decreased from 776 to 157 nm, as shown in Figure 2 and Table 2. We believe that these thickness changes were also due to the reduction in the bottom c-plane of GaNE.

In both cases, beside voids between S3-GaN and B2-GaN (circled with dashed lines), voids were also found between B3-GaN and B2-GaN (squared with dashed lines). These voids were created during the coalescence of GaN crystals [17].

### 4.3. Bottom C-Plane Protection

**Figure 5:**
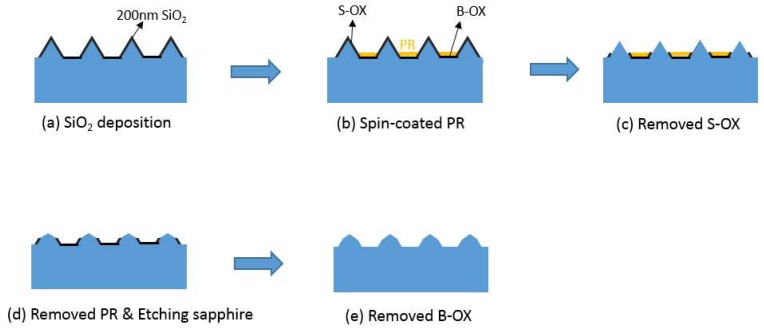
The flow charts of PSSO fabrication processes.

To avoid the reduction of the bottom c-plane areas of micron-sized PSS, the bottom c-plane was protected by SiO_2_ and then etched with sulfuric–phosphoric acid. This was designated as PSSO. Figure 5 shows the PSSO fabrication processes. Micron-sized RPSS was first deposited with 200-nm-thick SiO_2_ film (Figure 5a). A photoresist (PR) layer was spun onto the surface to protect the bottom oxide (B-OX). Sidewall oxide (S-OX) and PR were then removed, as shown in Figure 5b,c. Samples were etched in sulfuric–phosphoric acid at 270 °C for 30 s. B-oxide was then removed (Figure 5e).

Figure 4c is the high magnification of the PSSO surface. Only sidewall 3T facets were found, and no 6B facets were observed. Compared with RPSS, B3 fraction and B2 fraction were the same as those of RPSS. There was no reduction in the bottom c-plane areas of micron-sized PSSO.

Two kinds of PSSO samples were then fabricated to investigate the growth mechanism of GaN: (1) AlNOE (PSSO with AlN NL) and (2) GaNOE (PSSO with GaN NL). 

**Figure 6:**
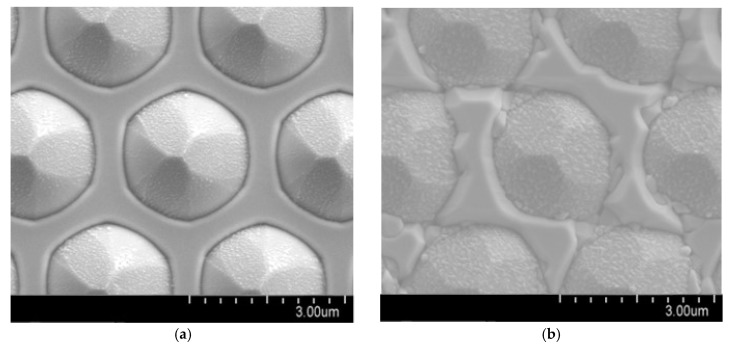
SEM images of GaN grown on (**a**) AlNOE and (**b**) GaNOE.

**Figure 7:**
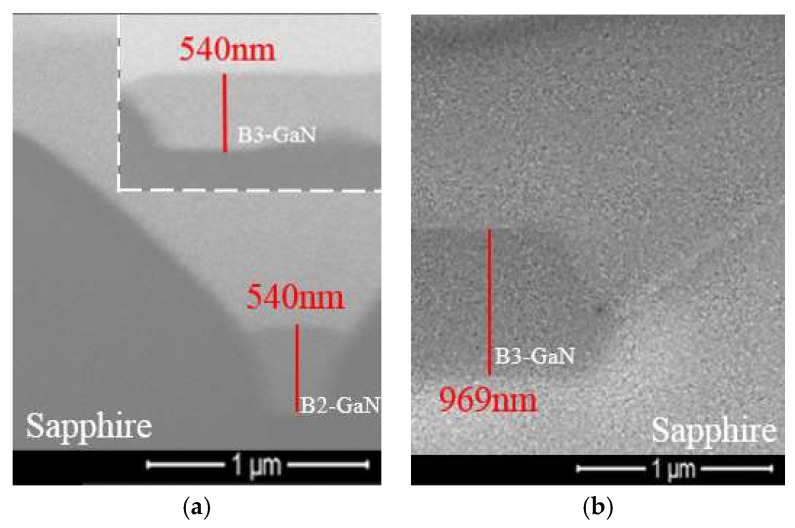
Cross-sectional SEM images from Figure 6. (**a**) AlNOE and (**b**) GaNOE.

As shown in Table 2 and Figure 6, no S3-GaN was grown from AlNOE, and no S6-GaN was grown from GaNOE either. In both case, no void was found among GaN crystals, as shown in Figure 7.

## 5. Conclusions

In this study, the growth of sidewall GaN was successfully suppressed by modifying the surface of micron-sized PSS. Sulfuric–phosphoric acid was used to modify the surface of dry etching c-plane PSS. Two kinds of nucleation layers—ex-situ AlN NL and in-situ GaN NL—were introduced prior to growth of GaN epilayer. 

After etching, three 3T {1$\bar{1}$05} facets were found on the pattern sidewall. At the same time, six 6B {3$\bar{4}$17} facets were observed on the bottom of the patterns. The appearance of 6B facets reduced the bottom c-plane fraction, which made epitaxy of GaN on bottom c-plane very difficult. Consequently, instead of reducing the growth of sidewall GaN, this modification appeared to enhance the growth of GaN from the sidewall of the patterns.

A 200-nm-thick SiO_2_ film was used to protect the bottom c-plane areas. After etching, only sidewall 3T facets were observed, and no 6B facet appeared. The bottom c-plane areas did not reduce. As a result, sidewall GaN was successfully suppressed in both NL systems.

## Figures and Tables

**Table 1:** Summary of sample preparation parameters.

Sample	AlNR	AlNE	GaNR	GaNE	AlNOE	GaNOE
Nucleation layers (NL)	AlN	AlN	GaN	GaN	AlN	GaN
PSS substrate	RPSS	PSSE	RPSS	PSSE	PSSO	PSSO

**Table 2:** The measured maximum thicknesses of GaN.

Thickness	GaN Type	AlNR	AlNE	AlNOE	GaNR	GaNE	GaNOE
Thickness (nm)	H_B3-GaN_	520	136	540	1261	616	969
	H_B2-GaN_	520	54	540	776	157	951
	H_S3-GaN_	74	250	0	0	0	0
	H_S6-GaN_	0	0	0	371	641	0
